# Enhanced Expansion and Sustained Inductive Function of Skin‐Derived Precursor Cells in Computer‐Controlled Stirred Suspension Bioreactors

**DOI:** 10.5966/sctm.2016-0133

**Published:** 2016-09-09

**Authors:** Natacha A. Agabalyan, Breanna S. Borys, Holly D. Sparks, Kathryn Boon, Eko W. Raharjo, Sepideh Abbasi, Michael S. Kallos, Jeff Biernaskie

**Affiliations:** ^1^ Department of Comparative Biology and Experimental Medicine, Faculty of Veterinary Medicine, University of Calgary, Calgary, Alberta, Canada; ^2^ Pharmaceutical Production Research Facility, Schulich School of Engineering, University of Calgary, Calgary, Alberta, Canada; ^3^ Department of Chemical and Petroleum Engineering, Schulich School of Engineering, University of Calgary, Calgary, Alberta, Canada; ^4^ Biomedical Engineering Graduate Program, University of Calgary, Calgary, Alberta, Canada; ^5^ Alberta Children's Hospital Research Institute, Calgary, Alberta, Canada; ^6^ Hotchkiss Brain Institute, Calgary, Alberta, Canada

**Keywords:** Dermal stem cells, Hair follicle, Bioprocess engineering, Mesenchyme, Regeneration, Scale‐up

## Abstract

Endogenous dermal stem cells (DSCs) reside in the adult hair follicle mesenchyme and can be isolated and grown in vitro as self‐renewing colonies called skin‐derived precursors (SKPs). Following transplantation into skin, SKPs can generate new dermis and reconstitute the dermal papilla and connective tissue sheath, suggesting they could have important therapeutic value for the treatment of skin disease (alopecia) or injury. Controlled cell culture processes must be developed to efficiently and safely generate sufficient stem cell numbers for clinical use. Compared with static culture, stirred‐suspension bioreactors generated fivefold greater expansion of viable SKPs. SKPs from each condition were able to repopulate the dermal stem cell niche within established hair follicles. Both conditions were also capable of inducing de novo hair follicle formation and exhibited bipotency, reconstituting the dermal papilla and connective tissue sheath, although the efficiency was significantly reduced in bioreactor‐expanded SKPs compared with static conditions. We conclude that automated bioreactor processing could be used to efficiently generate large numbers of autologous DSCs while maintaining their inherent regenerative function. Stem Cells Translational Medicine *2017;6:434–443*

**Significance Statement**

Recent work has demonstrated the existence of dermal stem cells (DSCs) that reside within adult hair follicles and might serve as a renewable source of inductive dermal cells to regenerate dermis or rejuvenate dermal papilla to restore follicle growth. In order to realize this clinical potential, it is essential that in vitro bioprocesses are developed to rapidly and safely expand DSCs. The present study showed that computer‐controlled stirred suspension bioreactors can be used to efficiently and safely generate large numbers of DSCs while maintaining their phenotype and at least some of their inherent inductive function. This builds on a foundation of research supporting automated bioprocessing as an effective approach for culturing stem cells. The bioprocess described has important implications for ex vivo expansion of inductive dermal stem/progenitor cells that could be used for composite skin engineering strategies, drug screens related to hair growth, and the regeneration of dermis within severe skin wounds.

## Introduction

The adult hair follicle (HF) is composed of mesenchymal cells that provide instructive signals to regulate epithelial stem cell function during homeostasis and tissue regeneration. Loss or dysfunction of the inductive mesenchyme residing within the dermal papilla prevents the onset of anagen hair growth and leads to hair loss 1.

Recent work has demonstrated the existence of a self‐renewing dermal stem cell (DSC) within the adult mammalian skin 2, 3. DSCs reside specifically in the HF mesenchyme and function to replenish the dermal papilla and connective tissue sheath (CTS) with each new regenerative hair cycle. DSCs can be isolated in vitro (referred to as skin‐derived precursors or “SKPs”), where they exhibit long‐term self‐renewal and can be persuaded to generate both mesodermal and neural derivatives 2, 4, 5, 6, 7, 8. When SKPs are transplanted to full‐thickness skin wounds, they give rise to a variety of fibroblast phenotypes and fill the lesion with new dermal tissue 2. Transplanted SKPs are also able to integrate into the mesenchyme of existing HFs and initiate formation of new HFs when cotransplanted with neonatal epithelial cells 2.

Thus, DSCs (or SKPs) represent a potentially valuable source of multipotent, self‐renewing dermal progenitors that could be used to repopulate the HF mesenchyme or the dermis after skin injury or disease. Like many stem cell cultures, SKPs are typically grown in static tissue culture flasks as nonadherent, self‐renewing spherical colonies. Although sufficient to generate cells for experimental purposes, this approach is impractical for generating the large quantities of SKPs that would be required in an autologous clinical cell therapy to repopulate the HF mesenchyme or dermis to enhance wound healing. Not only are static tissue culture methods time and labor intensive, but also a requirement exists for manual handling and inherent cellular variation between flasks 9. Previous studies, using cell types that included murine embryonic stem cells 10, 11, human embryonic stem cells 12, multipotent adult progenitor cells from bone marrow 13, neural precursor cells 14, mesenchymal stem cells 15, and induced pluripotent stem cells 16 have all shown that stirred suspension bioreactors are an effective alternative for culturing stem cells. Stirred suspension bioreactors offer several advantages over static cultures, including reduced labor and operating costs, higher yield, greater cellular homogeneity, reduced space requirements, and increased cell density per volume 17. They also allow for precise monitoring and control of key process variables. Controlling the physiochemical environment (pH and dissolved gas), nutrient and metabolite concentrations, and growth factors to most closely recapitulate their in vivo niche 18 will be imperative for translation to clinical applications and establishing Current Good Manufacturing Process‐compatible bioprocesses 19. The shear stresses produced in stirred suspension bioreactors can stimulate intracellular pathways governing expansion. In vivo, the cells are exposed to a combination of biochemical and physical cues that regulate their function. Mechanical stimulation, in particular, shear stress ex vivo, is acknowledged as having the ability to enhance both proliferation and differentiation of stem cells, making the effect of physical forces important for tissue engineering 20.

In the present study, we first queried whether computer‐controlled, stirred suspension bioreactors could be used to enhance expansion of primary adult SKPs in vitro. Next, we tested whether their endogenous bipotency and HF inductive function could be sustained over multiple passages in bioreactors in vitro.

## Materials and Methods

### Adult Rat SKP Isolation

Rat SKPs (rSKPs) were isolated from the back skin of P30 male green fluorescent protein (GFP)‐expressing Sprague Dawley rats (Sadaaki Takagi Japan SLC, Hamamatsu, Japan, http://www.jslc.co.jp) after an overdose of sodium pentobarbital (27.3 mg/kg, intraperitoneal injection), according to previously described protocols 21. Primary dermal cells were grown in basic fibroblast growth factor (40 ng/ml; BD Biosciences, San Jose, CA, http://www.bdbiosciences.com), platelet‐derived growth factor (PDGF)‐BB (25 ng/ml; R&D Systems, Minneapolis, MN, http://www.rndsystems.com), B27 supplement (2%; Thermo Fisher Scientific Life Sciences, Waltham, MA, http://www.thermofisher.com) and penicillin/streptomycin (1%; Thermo Fisher Scientific Life Sciences) in Dulbecco's modified Eagle's medium (DMEM) low glucose/F12 (3:1; Thermo Fisher Scientific Life Sciences). SKPs were plated at 30,0000 cells per milliliter in suspension culture flasks (Falcon). Following primary colony formation, SKPs were dissociated to single cells using collagenase digestion (2 mg/ml; Worthington Biochemical Corp., Lakewood, NJ, http://www.worthington‐biochem.com) and replated at 30,000 cells per milliliter. SKPs were passaged three or four times in static culture before being introduced to the bioreactor expansion to obtain sufficient numbers of cells to seed into the 500‐ml bioreactors at 30,000 cells per milliliter.

### Serial Passaging

**Figure 1:**
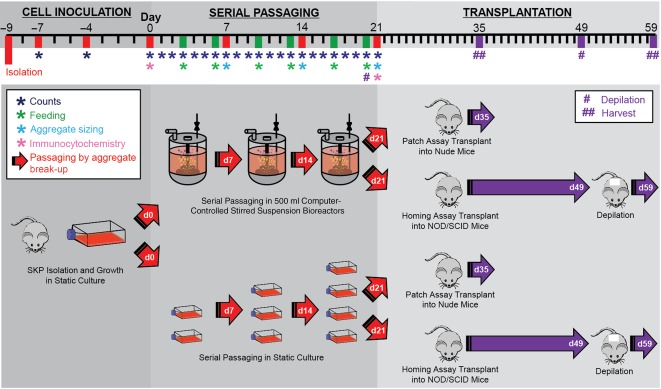
Experimental timeline for cell inoculation, serial passaging in bioreactor and static conditions, and patch assay and homing assay transplantations. Abbreviations: d, day; NOD/SCID, nonobese diabetic/severe combined immunodeficiency; SKP, skin‐derived precursor.

The overall experimental design is shown in Figure 1. rSKPs from each animal were cultured for three consecutive passages in 500‐ml computer controlled DASGIP Parallel Bioreactor Systems (Eppendorf, Hamburg, Germany, http://www.eppendorf.com) and, in parallel, in T75 suspension culture flasks (Cellstar; Greiner Bio One, Kremsmünster, Austria, http://www.gbo.com). For all passages, the variable bioreactor set points were regulated at 60 rpm, 37°C, pH 7.4, and a 21% dissolved oxygen concentration. rSKPs in static culture flasks were grown in a humidified incubator at 37°C and 5% CO_2_ in air. rSKPs were inoculated at 30,000 cells per milliliter and cultured for 7 days. On day 7 of each passage, rSKP aggregates were dissociated using collagenase digestion and counted by standard trypan blue exclusion. The cells were fed on days 3 and 6 of each passage with the growth factors B27 and penicillin/streptomycin. DASGIP Parallel Bioreactor Systems (Eppendorf) were taken offline to allow each passaging and feeding procedure to be conducted in a sterile biosafety cabinet.

### Analysis of Cell Growth Kinetics

Cell counts were performed at day 7 to calculate the multiplication ratios and cumulative multiplication ratios at each passage. Multiplication ratios were defined as the final viable cell density divided by the inoculation density (30,000 cells per milliliter). Cumulative multiplication ratios were defined as the product of the individual passage multiplication ratios for that culture condition.

To analyze the exponential doubling times and apparent growth rates in the DASGIP Parallel Bioreactor System (Eppendorf), cell counts were performed each day. Two samples of 2.0 ml were collected through a one‐way valve sample port using a 3‐ml manual screw dispensing syringe. Each sample was dissociated and counted twice. The results were used to generate viable cell density growth curves. The exponential phase doubling time and the apparent growth rate over the course of each passage were determined using Equations 1 and 2:

(1) $$X_{2}=X_{1}e^{-\mu \Delta t}$$

(2) $$t_{D}=\ln\left(2\right)/\mu$$

where *X* is the viable cell density at a given time point (cells per milliliter), *µ* is the growth rate (h^−1^), *t* is the time in hours, and *t_D_* is the population doubling time in hours.

The exponential doubling time (*t_D_*) was defined as the time required for the cell population to double in size during exponential growth (*X_1_*, *X_2_*, and *Δt* = *t_2_* − *t_1_* chosen during exponential phase), following the lag phase. The apparent growth rate (*µ*_App_) related the change in cell concentration from the inoculation density (*X_1_ = X_0_*) to the final exponential growth concentration (*X_2_ = X_F_*) over the entire passage (*Δt = t_F_ − t_0_*).

### Assessment of Aggregate Size

An aggregate was defined as a group of cells >40 μm in diameter. For both bioreactor and static culture conditions, the aggregate diameter was measured for 60 aggregates (*n* = 20 per animal, 3 animals for each condition) at the end of each serial passage. Aggregate size was determined by taking multiple 1.0‐ml samples to be imaged using a Zeiss Axiovert 25 microscope (Carl Zeiss Microscopy, Thornwood, NY, http://www.zeiss.com).

### Homing Assay

GFP expressing rSKPs grown in either bioreactor or static cultures were dissociated as described. One million cells were resuspended in 30 μl of DMEM and injected intradermally in the back skin of 4‐week‐old male nonobese diabetic/severe combined immunodeficiency mice, as described previously 2. One static rSKPs and one bioreactor rSKPs graft were included on each mouse. The back skin was depilated after 1 month to reset anagen growth and assess the fate of SKPs over successive cycles. Ten days later, the mice were euthanized. The transplant site was identified by GFP labeling and collected and fixed in 4% paraformaldehyde (PFA).

### Ex Vivo HF Formation Assay

HF formation assays were performed as previously described 2, 22. Four‐week‐old male nude mice (Nu/Nu) were anesthetized and injected subcutaneously with rSKP/keratinocyte suspension (15,000 epithelial aggregates combined with 10^6^ SKPs in 80 μl DMEM). One injection with static‐grown rSKPs and one injection with bioreactor‐grown rSKPs were performed per mouse. The grafts were left in place for 2 weeks, after which the mice were euthanized by CO_2_ and the back skin was collected.

### Tissue Preparation and Analysis

The mice were given an overdose of sodium pentobarbital, and the back skin was removed and subsequently fixed overnight in 4% PFA at 4°C and then placed in increasing sucrose solutions at 4°C each overnight (10%, 20%, and 30% sucrose) before being snap frozen in blocks of OCT in liquid nitrogen‐cooled isobutane and stored at −80°C. The skin was sectioned at 30‐μm thicknesses using a Leica CM1950 cryotome (Leica Biosystems, Buffalo Grove, IL, http://www.leicabiosystems) for immunohistochemical analysis (*n* = 24 sections per animal; 3 animals for each condition). The grafts were fixed overnight in 4% PFA at 4°C then changed to phosphate‐buffered saline (PBS) overnight at 4°C. The total number of GFP^+^ HFs was counted using a Zeiss Lumar V12 Stereo microscope, and higher magnification imaging was done with a Zeiss Observer microscope using Axiovision software (Carl Zeiss Microscopy).

### Immunofluorescence Staining

Bioreactor and static cell cultures were analyzed after three consecutive passages. The cells were immobilized on slides using a Cytospin (Shandon; Thermo Fisher Scientific Life Sciences) at 8,000 rpm for 5 minutes. The cells were blocked overnight at 4°C in 10% donkey serum/0.5% Triton‐X PBS. Primary antibodies were diluted in 1% donkey serum in PBS and incubated overnight at 4°C. Secondary antibodies were used at 1:500 in PBS and incubated overnight at 4°C.

The skin sections were incubated in 10% donkey serum/0.5% Triton‐X 100 in PBS for 2 hours at room temperature. Primary antibodies were diluted in 1% donkey serum PBS and incubated overnight at 4°C. Secondary antibodies were used at 1:500 in PBS and incubated for 2 hours at room temperature. Nuclei were labeled using Hoechst at 1:1,000, and the slides were coverslipped using Permafluor mounting medium (Thermo Fisher Scientific Life Sciences).

### Flow Cytometry

Bioreactor and static cell cultures were analyzed after three consecutive passages. rSKP spheres were centrifuged at 600*g* for 5 minutes, and the supernatant was removed and discarded. Cells were dissociated to single cells, fixed in 4% PFA at room temperature for 30 minutes before being centrifuged again, resuspended in 2 ml of PBS, and kept at 4°C. The cells were blocked for 30 minutes at room temperature in 10% donkey serum/0.5% Triton X PBS. Primary antibodies were diluted in 1% donkey serum in PBS and incubated for 30 minutes at room temperature. Secondary antibodies were used at 1:100 in PBS and for 30 minutes at room temperature. SKPs were analyzed with a FACS ARIA III sorter (BD Biosciences); 10,000 events were collected for each antibody of interest, and gates were set according to unstained and secondary only controls (in the absence of isotype controls). Cytometry data presentation was done with FLoJo (Tree Star, Ashland, OR, http://www.flowjo.com).

### Statistical Analysis

All statistical analyses were undertaken using GraphPad Prism, version 6.0 (GraphPad, San Diego, CA, http://www.graphpad.com). A two‐tailed Student *t* test or two‐way analysis of variance was used, followed by Sidak's multiple comparison test. Cell counts and aggregate sizing were pooled for each passage from 3 mice. Differences were considered statistically significant at *p* < .05. All in vitro graphs are presented as mean ± SEM.

## Results

### Computer Controlled Bioreactors Enhance Expansion of rSKPs Over Multiple Passages Relative to Static Culture

**Figure 2:**
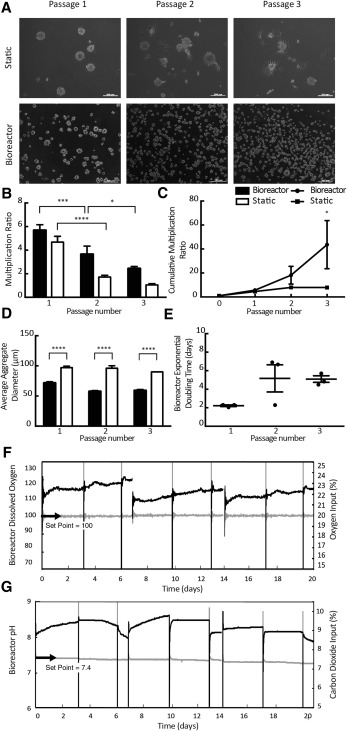
Rat skin‐derived precursor (rSKP) cells grown in computer‐controlled bioreactors produce significantly greater numbers of viable cells over multiple passages. **(A):** Representative images showing rSKPs continue to grow as aggregates over three consecutive passages in static and bioreactor conditions. **(B):** Multiplication ratios over serial passages in static and bioreactor conditions. A significant difference was found in the multiplication ratios between passages 1 and 2 in bioreactor and static conditions (*p* < .0001) and between passages 2 and 3 in the bioreactor condition (*p* < .05). By the third serial passage, a significant difference was found in the cumulative multiplication ratios between the bioreactor and static conditions (*p* < .05). **(C):** Cumulative multiplication ratios over serial passages in static and bioreactor conditions. At the end of each serial passage, the average aggregate size in the bioreactor condition was significantly smaller than in the static condition (*p* < .0001). **(D):** Average aggregate size of rSKP cells in static and bioreactor conditions. **(E):** Exponential doubling times in static and bioreactor conditions. **(F, G):** The bioreactor condition was under continuous feedback control regulating the dissolved oxygen concentration in the medium at 21% and the pH at 7.4. Scale bars = 200 μm. A two‐way analysis of variance (ANOVA) followed by Sidak's multiple comparison test was used for **(B–D)**, and a one‐way ANOVA followed by Sidak's multiple comparison test was used for **(E)**. Error bars represent SEM of three independent experiments. ****, *p* < .0001; ***, *p* < .001; *, *p* < .05.

SKP cells from GFP‐expressing rats (*n* = 3; rSKPs) were cultured in parallel static and bioreactor conditions for three consecutive passages (Fig. 1). After 7 days, aggregates were dissociated into single cells and reinoculated in static or bioreactor conditions (Fig. 2A).

Cell counts were performed daily for bioreactor culture and, on day 7, for static culture. The expansion of rSKP cells decreased significantly between serial passages 1 and 2 in bioreactor and static culture and between serial passages 2 and 3 in bioreactor culture (Fig. 2B). However, by the third consecutive passage, the total expansion of rSKP cells in bioreactor culture was significantly greater than in static culture (Fig. 2C). The average cumulative multiplication ratios at the end of the three serial passages in bioreactor and static conditions were 43.6 ± 20.1 and 7.91 ± 0.99 respectively, indicating a fivefold increase in overall expansion in the bioreactors.

Although the total expansion of rSKP cells was greater in bioreactor than in static culture, the average aggregate size of rSKP cells grown in bioreactor culture was significantly smaller than the average aggregate size of rSKP cells grown in static culture (Fig. 2D). These results were consistent for each passage. This difference is also qualitatively displayed in Figure 2A, in which a distinct difference in aggregate size can be seen between the day 7 images of the bioreactor and static cultures. The exponential doubling times in the bioreactors were calculated for each passage and the results pooled. No significant difference was seen between passages (Fig. 1E).

The DASGIP Parallel Bioreactor System (Eppendorf) allows for continuous feedback control over the dissolved oxygen concentration and the pH of the medium. Over the three serial passages, the bioreactor cultures were regulated at 21% dissolved oxygen concentration (Fig. 2F). The input oxygen percentage in the headspace gas increased in a linear fashion between days 1 and 7 of each passage. The average inoculation pH of the rSKP culture proliferation medium was 7.69 ± 0.03 at the beginning of each passage. The pH was regulated at 7.4 over each serial passage by the addition of carbon dioxide to the headspace gas (Fig. 2G). As a result of the high initial pH, the DASGIP Parallel Bioreactor System (Eppendorf) increased the input percentage of carbon dioxide to the system during the first half of each passage to reduce the pH to 7.4. The carbon dioxide input percentage then began to decrease during the second half of each passage as the cell numbers expanded, producing lactic acid and lowering the pH of the culture.

### Bioreactor Expansion Does not Change rSKP Characteristic Protein Expression

**Figure 3:**
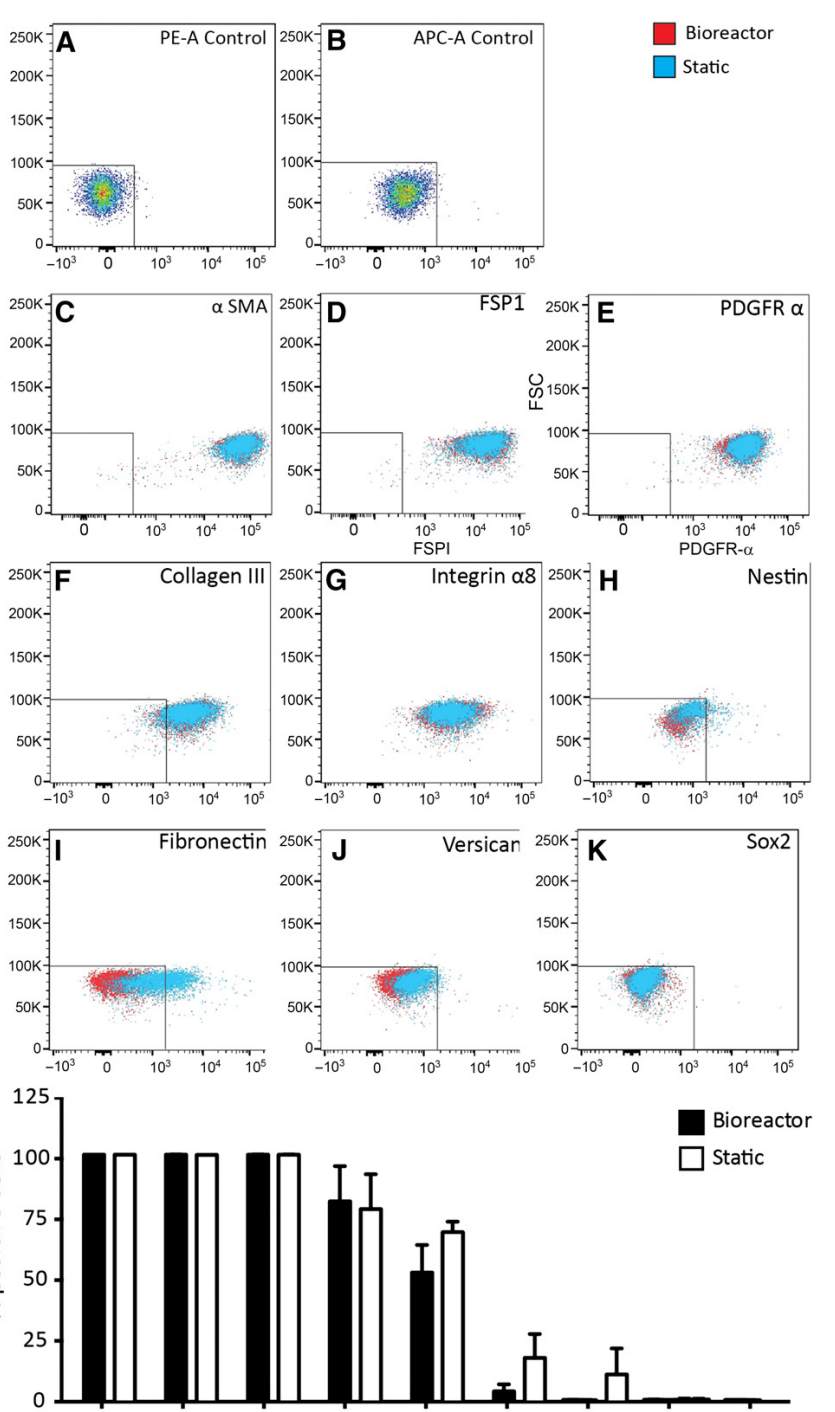
Characteristic skin‐derived precursor (SKP) markers in bioreactor versus static culture. **(A–K):** Flow cytometry of single‐cell rat SKPs grown for three consecutive passages under static and bioreactor conditions was performed to assess the protein expression of characteristic SKP markers. **(L):** No significant difference was found in the percentage of positive cell expression between the static and bioreactor cultures. Two‐tailed *t* test, *p* = nonsignificant. Error bars represent SEM of three independent experiments. Abbreviations: APC‐A, allophycocyanin; FB, fibronectin; FSC, forward scatter; FSP1, fibroblast‐specific protein 1; PDGFR‐α, platelet‐derived growth factor receptor‐α; PE‐A, r‐phycoerythrin; α‐SMA, α‐smooth muscle actin.

Following culture in both static and bioreactor conditions for three consecutive passages, rSKP marker expression was assessed by flow cytometry and immunofluorescence (representative static immunolabeling shown in the supplemental online data and supplemental online Fig. 1). rSKPs grown in the bioreactor culture showed a similar expression profile to rSKPs grown in static culture (Fig. 3). α‐Smooth muscle actin (α‐SMA), which marks DSCs and their progeny residing in the CTS, was maintained over successive passages and in both culture systems (Fig. 3C). Other DSC markers, fibroblast‐specific protein 1 (FSP1; Fig. 3D), PDGF receptor‐α (PDGFRα; Fig. 3E), and dermal extracellular matrix protein collagen III (Fig. 3F), were also maintained in both culture systems. Expression of the dermal papilla (DP)‐specific markers integrin α8 (Fig. 3G) and versican (Fig. 3J) were both maintained over multiple passages in both bioreactor and static expansion. SOX2, which is robustly expressed in the DP and DSCs contained within the mesenchyme of awl, auchene, and guard hair types, was undetectable after three passages in both conditions (Fig. 3K). A subset of SKPs from both conditions retained nestin expression and exhibited similar frequencies in both culture systems (Fig. 3H). Although fibronectin was expressed in a subset of both static and bioreactor expanded rSKPs, static cultures generated SKPs with elevated levels of fibronectin (Fig. 3I). Taken together, we found little phenotypic differences in SKPs exposed to either static or automated bioreactor expansion conditions (Fig. 3L) and most DSC markers, with the exception of SOX2, were sustained over multiple passages.

### Serial Bioreactor‐Expanded rSKPs Maintain Their Ability to Reconstitute the HF Mesenchyme and Induce HF Formation

**Figure 4:**
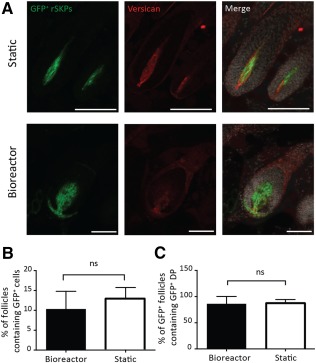
rSKPs repopulate the hair follicle (HF) mesenchyme after either bioreactor or static culture. **(A):** Representative images of endogenous HFs containing transplanted GFP^+^ rSKPs (green) from static and bioreactor grown rSKPs. Skin sections were immunostained for versican (red). **(B):** Percentage of GFP^+^ HFs. **(C):** Percentage of GFP^+^ dermal papilla HFs between static and bioreactor culture conditions. Scale bars = 100 μm. Two‐tailed *t* test, *p* = ns. Error bars represent SEM of three independent experiments. Abbreviations: DP, dermal papilla; GFP^+^, green fluorescent protein positive; ns, not significant; rSKP, rat skin‐derived precursor.

Our previous work demonstrated that SKPs are able to give rise to DP and CTS cell types and participate in HF morphogenesis 3. Therefore, we asked whether bioreactor expansion altered these functions. Following static or bioreactor culture, rSKPs were injected into the skin and then assessed for their ability to migrate into and repopulate the HF mesenchyme. Adult rSKPs cultured grown in either condition were able to home back to existing HFs and repopulate the mesenchymal compartments (Fig. 4A), with GFP^+^ cells taking residence in the dermal cup (the putative DSC niche), the dermal papilla, and the connective tissue sheath, suggesting that bipotency is retained. Quantification of three different transplants per group revealed that the total number of HFs containing donor GFP^+^ cells was unaffected by the growth conditions (13.01 ± 2.733; 8.858 ± 4.124; Fig. 4B). Similarly, no significant difference was found in the fate of donor SKPs within the hair follicle niche; that is, the percentage of those follicles containing GFP^+^ cells in the DP (86.11 ± 13.89; 87.67 ± 6.633; Fig. 4C) costained with DP marker versican did not differ between static versus bioreactor expansion conditions.

**Figure 5:**
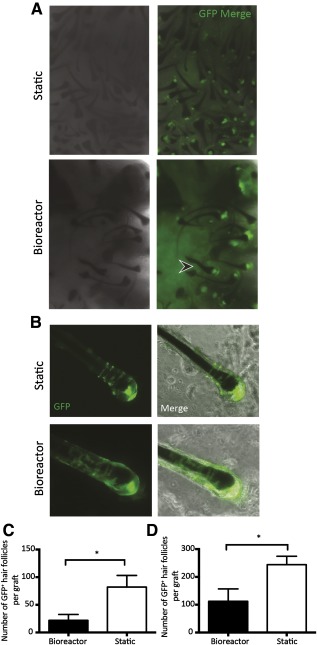
GFP rat skin‐derived precursors (rSKPs) from bioreactor and static culture retain their capacity to induce hair follicle (HF) morphogenesis. **(A):** Representative low‐magnification images of grafts containing bioreactor or static cultured GFP^+^ rSKPs combined with keratinocytes after 14 days. **(B):** Individually dissected HFs showing integration of GFP^+^ cells in the dermal papilla (DP) and the connective tissue sheath. **(C):** Number of GFP^+^ HFs formed within grafts containing either static‐ or bioreactor‐generated SKPs. Two‐tailed *t* test, *p* = .023. **(D):** Quantification of HF formation in the patch assay, with inclusion of wild‐type dermal fibroblasts to improve survival of donor cells. Note, despite supplementing each graft, bioreactor‐expanded GFP^+^ cells showed a sustained impairment. Error bars represent SEM of three independent experiments. Image magnifications are ×5 **(A)** and ×20 **(B)**. Abbreviation: GFP, green fluorescent protein.

We then studied whether bioreactor expansion affected their endogenous inductive. Using the patch assay, we transplanted donor SKPs grown in either condition, with GFP^NEG^ epithelial keratinocytes. Two weeks later, subcutaneous grafts yielded newly formed HFs, and most (>95%) of follicles within each graft contained a GFP^+^ DP, and only these follicles were counted, because our interest was in ascertaining the number of follicles induced by donor SKPs. Newly formed HFs from both conditions contained mesenchyme that was almost entirely composed of GFP^+^ cells, including the DP, dermal cup, and CTS (Fig. 5A, 5B), suggesting that expansion of inductive adult SKPs in stirred suspension bioreactor maintains bipotency and at least partially preserves inductive function. Although bioreactor‐grown SKPs retained their capacity to induce HF morphogenesis, they exhibited a significant reduction in hair forming ability compared with static‐expanded SKPs (*p* = .026), with nearly a twofold reduction in GFP^+^ HFs per graft (Fig. 5C). Grafts containing bioreactor‐expanded SKPs also showed a greater variation in follicle bulb size and many follicles exhibiting shorter hair fiber length (Fig. 5B, arrowhead), although this was not explicitly quantified. In a parallel set of experiments, we supplemented bioreactor or static SKPs with wild‐type neonatal dermal cells to enhance donor cell survival and potentially facilitate integration of bioreactor SKPs. Despite supplementing with dermal cells, bioreactor‐generated SKPs still exhibited impaired inductive capacity as shown by a marked reduction in GFP^+^ HF formation (*p* < .02; Fig. 5D). Taken together, stirred suspension bioreactors might serve as a valuable bioprocess platform for controlled (and automated) scale‐up of autologous dermal stem cells or inductive dermal papilla cells with the capacity to partially preserve inductive function even after five passages in vitro.

## Discussion

In the present study, the expansion, morphology, and growth kinetics of rSKP cells grown in static culture was compared with rSKP cells grown in computer‐controlled, stirred suspension bioreactor systems over consecutive passages. Thus, we were working to develop a bioprocess capable of producing clinically relevant numbers of SKP cells that maintain their functionality in a controlled and reproducible fashion.

We found that rSKP cells maintain their ex vivo phenotype over serial passages and continue to grow as suspended, spherical aggregates. In static culture, a subset of aggregates consistently adhered to the surface of the culture flask, and the frequency of adhesion appeared to increase over passages. All rSKP aggregates grown in the bioreactor remained in suspension, as the reactors were siliconized before use, thus preventing adhesion to the vessel surface. Based on our previous work 2, adhesion is indicative of differentiation and would largely contribute to the limited expansion observed in static culture. The average size of the rSKP cell aggregates on day 7 was significantly lower in the bioreactor conditions than in the static cultures. This was likely because of the shear stress present in the stirred suspension bioreactor, which can have direct and indirect effects. The optimal hydrodynamic environment will vary for different cell types and can be determined by comparing the growth kinetics at different agitation speeds. The shear stress present in the fluid is dependent on the bioreactor and impeller geometry, impeller agitation rate, cell density, and media viscosity 23. The maximum shear stress present at the tip of the impeller in the current bioreactors, operated at 60 rpm was 4.0 dyn/cm^2^, corresponding to a Kolmogorov eddy size of 150 μm. Particles (i.e., aggregates) larger than this size would be expected to have interactions and possible damage from shear. It is predicted that at a lower agitation rate, the average aggregate size in the bioreactor would increase as cells are less likely to shear apart 24. Moreover, we have shown that shear in stirred suspension can play a role in the stem cell aggregate packing density 25 and expression of stem cell markers 26. The aggregate size can also influence the health of the culture, because nutrients must diffuse through multiple cell layers to reach cells at the center of the aggregates. However, the small aggregate sizes present in both the static and the bioreactor systems in this experiment should not pose any mass transfer resistances to oxygen and nutrient diffusion 27. However, cultured SKPs generate colonies that do not typically exceed 300 µm in diameter, suggesting that aggregate size (and hence proliferation) is intrinsically regulated. Exposure to shear force in stirred suspension bioreactors might liberate single cells from proliferating aggregates, thereby reducing the average colony size and allowing for formation of new colonies and producing an overall increase in viable cell number.

Stirred suspension bioreactors, in which culture parameters, including dissolved oxygen, pH, and fluid shear, are steadily controlled, provide a healthy environment for cells and often lead to increased cell proliferation. Agitation can be used to control the size of aggregates and prevent cells from settling on the bottom of the bioreactor. The agitation rate of 60 rpm was chosen for the present study based on previous results that found the agitation rate can directly influence the diameter of spherical cell aggregates in 100‐ml stirred suspension bioreactors 25. Although aggregates in the bioreactor conditions proved to be smaller than in static culture, the bioreactor was able to produce a significantly greater number of cells after three consecutive passages. In general, higher agitation rates lead to higher shear stresses and smaller aggregates in stirred suspension bioreactors 10. An increased expansion of stem cells in computer‐controlled bioreactor systems for large‐scale production has also been documented for mesenchymal stem cells 28. Although the agitation rate in the present study was not optimized, it was sufficient to maintain the aggregates in suspension, maintain high viabilities, and expand the cells.

Stirred suspension bioreactors, as a scalable method for cell expansion, although offering a well‐mixed delivery of oxygen and nutrients to the cells, also change the geometry and physical environment of the cells and cell colonies. For example, we have shown, for pluripotent stem cells, that gene expression can be influenced by the shear stress present in the bioreactors 26. Although outside the scope of the present study, more detailed studies, both computational and experimental, on the effects of this dynamic environment on the function and population growth kinetics of cells, are necessary before truly robust clinical cell production systems can be put into widespread use.

The growth kinetic parameters of rSKP cells grown in the bioreactors did not differ greatly between passages. No significant difference was found in the exponential doubling times and no significant difference was seen in the apparent growth rates between passages. The data from one animal appear inconsistent with the data from other animals at passage 2, having a smaller exponential doubling time and greater apparent growth rate. By passage 3, however, the kinetic parameters for all animals were consistent again. Such variations can be attributed to donor variability.

The DASGIP Parallel Bioreactor System (Eppendorf) provided a controlled system for the expansion of rSKP cells by regulating the agitation rate, temperature, dissolved oxygen concentration, and pH. This feedback control is extremely important in creating a reproducible process, especially when managing patient‐to‐patient variability. If cells from a donor expand at a greater rate, typically more oxygen will be consumed and more lactic acid will be produced, changing the dissolved oxygen concentration and the pH of the culture. In the DASGIP Parallel Bioreactor System (Eppendorf), the culture was regulated at 21% dissolved oxygen and 7.4 pH to mimic the environment of the human body. Generally, cell culture incubators are set to input 21% oxygen and 5% carbon dioxide. The DASGIP Parallel Bioreactor System (Eppendorf) was successful in regulating the dissolved oxygen concentration and pH of the rSKP culture during each passage. The DASGIP system does not measure the nutrient levels within the culture; however, regulated feeding can be set up to allow glucose and growth factors to be fed into the reactor at controlled rates. Future work is required to determine the consumption rates specific to rSKP cells and the optimal nutrient and growth factor concentrations within the medium.

To expand SKPs in bioreactors for clinically relevant purposes, it is important for the cells to maintain their phenotype and HF inductive function. General dermal fibroblast markers (α‐SMA, FSP1, PDGFR α, collagen III) were similarly expressed after bioreactor or static expansion. Versican, an extracellular matrix protein found specifically in the inductive mesenchyme the dermal papilla and connective tissue sheath, was notably absent (or extremely low) from both populations. Despite this, we found both rSKP populations maintained their homing and inductive capacity in contrast to previous reports showing a strong association between versican expression and inductive capacity 29. Integrin‐α8, which is enriched in the HF mesenchyme and plays a role in cell‐cell adhesion and cell signaling, was sustained in rSKPs grown in both conditions. An increased expression of fibronectin was noted in the cells grown under bioreactor conditions, which could be due to stronger cell‐cell adhesion properties under stirred suspension conditions. SOX2 was not detectable by immunofluorescence after growth of rSKPs in either condition. However, this did not appear to influence their capacity to reconstitute the DP of established HFs or their inductive capacity for de novo HF morphogenesis. This is in line with recent work in which *Sox2* was deleted specifically in DP and failed to elicit an effect on inductive function during HF morphogenesis 30. We have previously noted that SKPs isolated from Sox2:GFP mice also exhibited a loss of GFP expression after several passages in vitro; however, this was restored after transplantation and reintegration into the HF DP 2. This suggests that factors within the HF niche might induce Sox2 expression in DP and DSCs but is not required for inductive DP function 30. Additional work is required to determine whether Sox2 might play a distinct role in DSCs.

SKPs grown under both conditions maintained their inductive capabilities and ability to home back to the HF dermal stem cell niche. However, inductive function was partially diminished after expansion in stirred suspension bioreactors. Previous work has demonstrated that the aggregation of DP cells results in sustained inductive function relative to monolayer culture 31. Our data support that idea but also suggest that other factors could contribute to inductive function. The aggregate size might allow for important cellular interactions that underlie inductive function, much like that reported for the DP in vivo 32. Recent in vivo work has suggested that a minimum number of cells are required to sustain normal inductive functions within the adult dermal papilla. One interpretation of our data is that similar cellular thresholds appear to govern the inductive capacity of SKPs such that smaller aggregates generated in the bioreactor exhibit different functional properties. Biophysical factors such as shear stress might activate and inadvertently bias the differentiation of SKPs toward a committed connective tissue sheath fate as opposed to acquisition of a DP phenotype. Alternatively, changes might occur in extracellular matrix production and composition within the smaller aggregates that are critical mediators of their inductive function. Future studies will consider the transcriptional status of cells grown in either condition to better understand the limited inductive capacity after bioreactor expansion and in identifying the inductive signals that mediate this process. That our data show that inductive function is partially retained following extensive cell scale‐up is promising. Ongoing experiments will examine additional media supplements that could be used to sustain inductive function. Moreover, equivalent shear stress has been shown to modulate *Sox2* and other pluripotency genes in embryonic stem cells 26. Therefore, activation of “stem cell” genes within DSC/SKPs that promote proliferation and prevent differentiation might consequently limit the acquisition of an inductive DP signature 33. Nevertheless, our data suggest that the inductive function of adult SKPs is mostly preserved after bioreactor expansion. Further optimization of the bioreactor system (agitation rate, impeller size, volume of culture medium), promotion of aggregate size, and inclusion of additional factors are needed to sustain the inductive capabilities of SKPs grown in bioreactor conditions and increasing the rapid, controlled expansion of cells.

## Conclusion

Our results indicate that computer‐controlled stirred suspension bioreactors can be used for robust expansion of isolated inductive adult dermal stem cells (SKPs) and, even after multiple (*n* = 5) passages in vitro, are able to sustain at least a part of their inherent HF inductive capacity and their ability to repopulate the mesenchyme of pre‐existing follicles (their endogenous niche). Five times as many cells were obtained in the bioreactor cultures compared with static cultures; thus, using bioreactor expansion early after isolation might better preserve inductive function, without limiting the cell yield in a contained and controlled environment. Overall, this approach might provide a safe and efficient method to generate large numbers of DSCs, with greater homogeneity, thereby permitting drug screening for compounds that might influence HF growth or cell‐based strategies to repopulate the skin and hair follicle after injury or disease.

## Author Contributions

N.A.A. and B.S.B.: conception and design, collection and/or assembly of data, data analysis and interpretation, manuscript writing; H.D.S.: collection and/or assembly of data, data analysis and interpretation; K.B.: collection and/or assembly of data, manuscript writing; E.W.R. and S.A.: collection and/or assembly of data; M.S.K.: conception and design, financial support, manuscript writing; J.B.: conception and design, financial support, manuscript writing, final approval of manuscript.

## Disclosure of Potential Conflicts of Interest

The authors indicated no potential conflicts of interest.
